# Editorial: Molecular and cellular mechanisms of the legume-rhizobia symbiosis, volume II

**DOI:** 10.3389/fpls.2023.1208904

**Published:** 2023-05-29

**Authors:** Jianping Wang, Catalina Iulia Pislariu, Cheng-Wu Liu, Viktor E. Tsyganov, Maitrayee DasGupta

**Affiliations:** ^1^ Agronomy Department, University of Florida, Gainesville, FL, United States; ^2^ School of the Sciences, Division of Biology, Texas Woman’s University, Denton, TX, United States; ^3^ School of Life Sciences, Division of Life Sciences and Medicine, University of Science and Technology of China, Hefei, China; ^4^ Laboratory of Molecular and Cellular Biology, All-Russia Research Institute for Agricultural Microbiology, Saint Petersburg, Russia; ^5^ Department of Biochemistry, University of Calcutta, Kolkata, India

**Keywords:** nitrogen (N), legume, symbiosis, rhizobium, root nodule, rhizobial infection

Nitrogen (N) fixation becomes increasingly imperative in sustainable crop production. Legumes form mutualistic symbiotic relationships with nitrogen-fixing rhizobia which fix substantial amounts of atmospheric N into ammonia inside root nodules, allowing legume plants to grow well in N-deficient soils, hereby eliminating N fertilizer application. The biologically fixed N by legumes accounts for about 65% nitrogen utilization in current global agriculture (Burris & Roberts, 1993). This legume-rhizobia symbiosis leads to the formation of a specialized new root organ, the nodule, which functions as a N-fixation factory, providing ideal conditions to accommodate large numbers of rhizobia inside host cells where they carry out nitrogen fixation. In nodules, rhizobia differentiate to bacteroids inside organelle-like symbiosomes (Coba de la Peña et al., 2018). Bacteroids obtain photosynthetic products from host plant and produce nitrogenase to fix atmospheric nitrogen into ammonia (Udvardi and Day, 1997). The host plant assimilates ammonia for growth and development, meanwhile produces the oxygen binding protein leghemoglobin to maintain the low oxygen environment necessary for nitrogenase function (Larrainzar et al., 2020).

Development of symbiotic nodules on legume roots is governed by a host genetic program that synchronizes two parallel processes, nodule organogenesis and bacterial infection (Guinel and Geil, 2002; Tsyganov et al., 2002), which means that when nodule primordia are formed from root cortical cells, a bacterial infection process coordinately targets the developing nodule primordia. Establishment of symbiosis is a multistep process requiring precisely timed signal exchange between the partners and a series of mutual accommodations. Up to date, over hundreds of genes have been functionally characterized for their roles in legume symbiosis (summarized by Roy et al., 2020; Tsyganov and Tsyganova, 2020) revealing a complex molecular mechanism of the symbiosis process. The purpose of this research topic was to put together the papers of new discoveries, perspectives, and overviews on the molecular mechanism of legume-rhizobia symbiosis. In this research topic, we collected two research articles and two review papers, covering functional genes during early rhizobial infection and amino acids and antioxidants’ functions in symbiosis establishment and development.

When the legume plant perceives the rhizobial or arbuscular mycorrhizal signal for symbiosis, Ca^2+^ oscillation is induced in the nuclei of infected cells, hereby activating calmodulin (CaM) and Ca^2+/^CaM-dependent protein kinase (CCaMK) to phosphorylate transcription factors and initiate downstream signaling events (reviewed by Oldroyd, 2013). In the review by Yuan et al., the molecular mechanisms underlying Ca^2+/^CaM-mediated signaling pathway in fine-tuning local and system symbiotic events were summarized, which can serve as an introduction for readers interested in the bacterial or fungal symbiosis to grasp the latest advances in Ca^2+/^CaM-mediated signaling for symbiosis establishment.

Multiple transcription factors have been identified to be involved in regulating establishment of root nodule symbiosis in model legume species *Mecicago truncatula* and *Lotus japonicus*, such as ERF Required for Nodulation1 (ERN1) in a transcription network with CYCLOPS and Nodule Inception (NIN) (Cerri et al., 2012; Cerri et al., 2017). To further clarify the roles of LjERN1 during root nodule symbiosis, Liu et al. compared the transcript profiles of wild-type *L. japonicus* and *Ljern1-6* mutants and reported that LjERN1 was involved in regulating multiple processes during the early establishment of root nodule symbiosis in coordination with LjNIN. The results extended our understanding of the pleiotropic role of LjERN1 in root nodule symbiosis.

Several earlier studies suggested that proline metabolism may play an essential role in legume-rhizobia symbiosis under stress. However, different or contradictory results were reported in several legume species. Sabbioni and Forlani summarized all the findings with a focus on an enzyme in a rate limiting step of proline synthesis and shed light on the emerging role of proline in the establishment and function of legume-rhizobium symbiosis.

Legume root nodule development is also linked to reactive oxygen species production. Thiol glutathione (GSH) is an antioxidant present in root nodules and functions as a redox buffer (Becana et al., 2010). Both GSH and its legume-specific homolog homoglutathione (hGSH) (one amino acid different from GSH) are involved in nodulation (Frendo et al., 2005). However, their exact functions in nodules are unknown. Ivanova et al. compared effective and ineffective pea symbiotic nodules to test the involvement of both thiols in nodule development and functioning, as well as in plant defense responses triggered by plant symbiosis-related mutations. The results revealed that certain level of thiols is required for proper symbiotic nitrogen fixation and the content or GSH:hGSH ratio changes are associated with different abnormalities and defense responses.

In summary, this research topic highlights a few recent discoveries, reviews, and emerging trends in deciphering new molecular mechanisms of legume-rhizobial symbiosis, which will pave the road toward advancing sustainable crop production and possibly introducing N fixation in non-legume crops.

## Author contributions

JW prepared the first draft of the editorial. CP, C-WL, VT, and MD critically revised and improved the draft. All authors approved the submitted version.
